# Methyl 1-{4-[(*S*)-2-(meth­oxy­carbon­yl)pyrrolidin-1-yl]-3,6-dioxocyclo­hexa-1,4-dien-1-yl}pyrrolidine-2-carboxyl­ate

**DOI:** 10.1107/S1600536810027947

**Published:** 2010-07-21

**Authors:** Simon J. Garden, Janet M. S. Skakle, Edward R. T. Tiekink, James L. Wardell

**Affiliations:** a Instituto de Química, Departamento de Quimica Orgânica, Universidade, Federal do Rio de Janeiro, Ilha do Fundão, CT, Bloco A, Rio de Janeiro 21949-900, RJ, Brazil; b Department of Chemistry, University of Aberdeen, Old Aberdeen AB15 5NY, Scotland; c Department of Chemistry, University of Malaya, 50603 Kuala Lumpur, Malaysia; d Centro de Desenvolvimento Tecnológico em Saúde (CDTS), Fundação Oswaldo Cruz (FIOCRUZ), Casa Amarela, Campus de Manguinhos, Av. Brasil 4365, 21040-900 Rio de Janeiro, RJ, Brazil

## Abstract

The complete mol­ecule of the title diproline ester quinone, C_18_H_22_N_2_O_6_, is generated by a crystallographic twofold axis, which passes through the centre of the benzene ring. Both –CO_2_Me groups are orientated to the same side of the benzene ring, with the carbonyl groups pointing roughly towards each other. The conformation of the proline residue is an envelope. In the crystal, a three-dimensional network is sustained by C—H⋯O inter­actions involving both the quinone and carbonyl O atoms.

## Related literature

For the oxidative nucleophilic addition of amines to quinones to form amino­quinones, see: Lyons & Thomson (1953 ▶). For background to mitomycin anti­cancer drugs, see: Tomasz (1995 ▶). For additional geometric analysis, see: Cremer & Pople (1975 ▶).
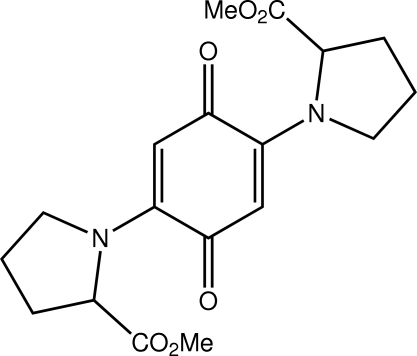


## Experimental

### 

#### Crystal data

C_18_H_22_N_2_O_6_
*M* *_r_* = 362.38
Monoclinic, 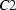

*a* = 11.4728 (5) Å
*b* = 7.1556 (4) Å
*c* = 11.7882 (7) Å
β = 111.230 (3)°
*V* = 902.07 (8) Å^3^
*Z* = 2
Mo *K*α radiation
μ = 0.10 mm^−1^
*T* = 120 K
0.24 × 0.12 × 0.08 mm

#### Data collection

Nonius KappaCCD area-detector diffractometer
Absorption correction: multi-scan (*SADABS*; Sheldrick, 2007 ▶) *T*_min_ = 0.896, *T*_max_ = 1.000
6840 measured reflections
1114 independent reflections
1008 reflections with *I* > 2σ(*I*)
*R*_int_ = 0.034

#### Refinement

*R*[*F* ^2^ > 2σ(*F* ^2^)] = 0.038
*wR*(*F* ^2^) = 0.136
*S* = 1.23
1114 reflections
119 parameters
1 restraint
H-atom parameters constrained
Δρ_max_ = 0.64 e Å^−3^
Δρ_min_ = −0.63 e Å^−3^

### 

Data collection: *COLLECT* (Hooft, 1998 ▶); cell refinement: *DENZO* (Otwinowski & Minor, 1997 ▶) and *COLLECT*; data reduction: *DENZO* and *COLLECT*; program(s) used to solve structure: *SHELXS97* (Sheldrick, 2008 ▶); program(s) used to refine structure: *SHELXL97* (Sheldrick, 2008 ▶); molecular graphics: *ORTEP-3* (Farrugia, 1997 ▶) and *DIAMOND* (Brandenburg, 2006 ▶); software used to prepare material for publication: *publCIF* (Westrip, 2010 ▶).

## Supplementary Material

Crystal structure: contains datablocks global, I. DOI: 10.1107/S1600536810027947/hb5550sup1.cif

Structure factors: contains datablocks I. DOI: 10.1107/S1600536810027947/hb5550Isup2.hkl

Additional supplementary materials:  crystallographic information; 3D view; checkCIF report

## Figures and Tables

**Table 1:** Hydrogen-bond geometry (Å, °)

*D*—H⋯*A*	*D*—H	H⋯*A*	*D*⋯*A*	*D*—H⋯*A*
C3—H3⋯O2^i^	0.95	2.56	3.400 (3)	147
C5—H5b⋯O1^ii^	0.99	2.54	3.407 (3)	146
C9—H9b⋯O1^iii^	0.98	2.38	3.186 (4)	139

Symmetry codes: (i) 
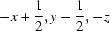
; (ii) 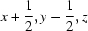; (iii) 
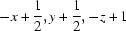
.

**Fig. 1.**
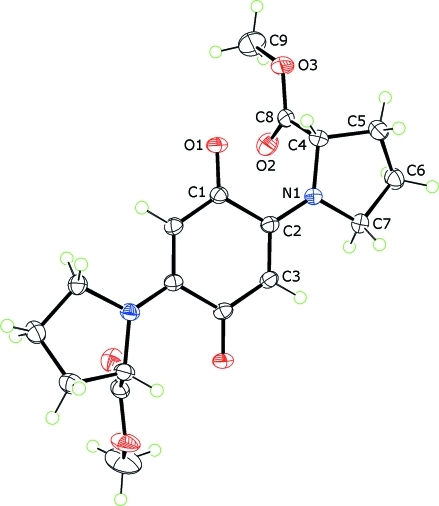
The molecular structure of (I) showing displacement ellipsoids at the 50% probability level. Unlabelled atoms are generated by (–x, y, –z).

**Fig. 2.**
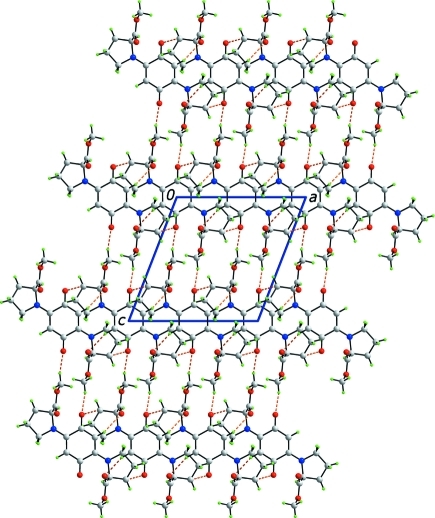
A view in projection down the b axis of the unit-cell contents (I) showing the C–H···O contacts as orange dashed lines.

## supplementary crystallographic information

### Comment

Oxidative nucleophilic addition of amines to quinones results in the formation of aminoquinone products (Lyons & Thomson, 1953). As part of a study into concise methodology for the synthesis of heterocyclic systems, we envisaged that oxidative addition of α-amino acid derivatives to benzoquinone could yield a suitably functionalized precursor for cyclization to yield pyrroloindole quinones, a structural motif present in the mitomycin anticancer drugs (Tomasz, 1995). The title diproline ester quinone, (I), was synthesized in this context.

The molecule of (I), Fig. 1, exists about a crystallographic 2-fold axis of symmetry passing through the centre of the benzene ring. This has the result that the two –CO_2_Me groups are orientated to the same side of the benzene ring. The carbonyl groups are tucked in under the benzene ring. The conformation of the proline residue is an envelope with the C5 atom lying above the plane through the remaining atoms. The conformational descriptors (Cremer & Pople, 1975) are Q(2) = 0.348 (3) Å and φ(2) = 79.3 (4) °.

The crystal packing features C—H···O contacts, Table 1. The quinone-O1 atom accepts two such interactions, one from a methylene-H and the other from a methyl-H, whereas the carbonyl-O2 accepts a quinone-H. The C—H···O interactions combine to give a 3-D network, Fig. 2.

### Experimental

Proline methyl ester hydrochloride (1.63 g, 9.8 mmol) and KOAc (1.07 g, 10.9 mmol) were mixed in MeOH (20 ml). Excess benzoquinone (1.00 g, 9.3 mmol) was added to the solution resulting in a deep-red coloured reaction. The reaction was stirred for an hour then all the volatiles were removed under reduced pressure. The crude product was solubilized in EtOAc and filtered through a plug of silica using EtOAc as eluent. The red coloured fraction was evaporated under reduced pressure and the product chromatographed on a column of silica eluting with CH_2_Cl_2_ (removed excess benzoquinone) followed by a CH_2_Cl_2_/EtOAc gradient (9:1 V/V to 4:1 V/V). Evaporation of the product containing fractions and recrystallization from MeOH gave 0.266 g of dark-red prisms of (I) (15% yield), m.pt. 463–465 K.

^1^H (CDCl_3_): δ 1.95 [2H, m]; 2.19 [2H, m]; 3.39 [1H, m]; 3.48 [1H, m]; 3.73 [3H, s]; 5.07 [1H, bs]; 5.37 [1H, bs] p.p.m. ^13^C (CDCl_3_): δ 22.0; 31.6; 51.3; 52.4; 62.9; 101.5; 148.7; 172.8; 181.2 p.p.m. IR (cm^-1^): 3066, 2982, 2955, 2927, 2881, 1744, 1625, 1555, 1432, 1349, 1274, 1207, 1166, 957, 833, 794. Mass (a.m.u.) abundance %: 362 (79), 331 (10), 303 (52), 276 (100), 261 (63), 243 (25), 235 (37), 217 (46), 176 (25), 122 (37).

### Refinement

The C-bound H atoms were geometrically placed (C–H = 0.95–1.00 Å) and refined as riding with *U*_iso_(H) = 1.2–1.5*U*_eq_(C). The maximum and minimum residual electron density peaks of 0.64 and 0.63 e Å^-3^, respectively, were located 1.59 Å and 0.85 Å from the H5b and C8 atoms, respectively. In the absence of significant anomalous scattering effects, 750 Friedel pairs were averaged in the final refinement. However, the absolute configuration was assigned on the basis of the chirality of the *L*-proline starting material.

### Crystal data

**Table:**

C_18_H_22_N_2_O_6_	*F*(000) = 384
*M**_r_* = 362.38	*D*_x_ = 1.334 Mg m^−^^3^
Monoclinic, *C*2	Mo *K*α radiation, λ = 0.71073 Å
Hall symbol: C 2y	Cell parameters from 1107 reflections
*a* = 11.4728 (5) Å	θ = 2.9–27.5°
*b* = 7.1556 (4) Å	µ = 0.10 mm^−^^1^
*c* = 11.7882 (7) Å	*T* = 120 K
β = 111.230 (3)°	Prism, dark-red
*V* = 902.07 (8) Å^3^	0.24 × 0.12 × 0.08 mm
*Z* = 2	

### Data collection

**Table:**

Nonius KappaCCD area-detector diffractometer	1114 independent reflections
Radiation source: Enraf Nonius FR591 rotating anode	1008 reflections with *I* > 2σ(*I*)
10 cm confocal mirrors	*R*_int_ = 0.034
Detector resolution: 9.091 pixels mm^-1^	θ_max_ = 27.5°, θ_min_ = 3.4°
φ and ω scans	*h* = −14→14
Absorption correction: multi-scan (*SADABS*; Sheldrick, 2007)	*k* = −8→9
*T*_min_ = 0.896, *T*_max_ = 1.000	*l* = −15→15
6840 measured reflections	

### Refinement

**Table:**

Refinement on *F*^2^	Primary atom site location: structure-invariant direct methods
Least-squares matrix: full	Secondary atom site location: difference Fourier map
*R*[*F*^2^ > 2σ(*F*^2^)] = 0.038	Hydrogen site location: inferred from neighbouring sites
*wR*(*F*^2^) = 0.136	H-atom parameters constrained
*S* = 1.23	*w* = 1/[σ^2^(*F*_o_^2^) + (0.0929*P*)^2^] where *P* = (*F*_o_^2^ + 2*F*_c_^2^)/3
1114 reflections	(Δ/σ)_max_ < 0.001
119 parameters	Δρ_max_ = 0.64 e Å^−^^3^
1 restraint	Δρ_min_ = −0.63 e Å^−^^3^

### Special details

**Table:**

Geometry. All s.u.'s (except the s.u. in the dihedral angle between two l.s. planes) are estimated using the full covariance matrix. The cell s.u.'s are taken into account individually in the estimation of s.u.'s in distances, angles and torsion angles; correlations between s.u.'s in cell parameters are only used when they are defined by crystal symmetry. An approximate (isotropic) treatment of cell s.u.'s is used for estimating s.u.'s involving l.s. planes.
Refinement. Refinement of *F*^2^ against ALL reflections. The weighted *R*-factor *wR* and goodness of fit *S* are based on *F*^2^, conventional *R*-factors *R* are based on *F*, with *F* set to zero for negative *F*^2^. The threshold expression of *F*^2^ > 2σ(*F*^2^) is used only for calculating *R*-factors(gt) *etc*. and is not relevant to the choice of reflections for refinement. *R*-factors based on *F*^2^ are statistically about twice as large as those based on *F*, and *R*- factors based on ALL data will be even larger.

### Fractional atomic coordinates and isotropic or equivalent isotropic displacement parameters (Å^2^)

**Table:**

	*x*	*y*	*z*	*U*_iso_*/*U*_eq_	
O1	0.08333 (15)	0.9788 (3)	0.24336 (14)	0.0259 (4)	
O2	0.27854 (17)	1.2823 (3)	0.25151 (17)	0.0334 (5)	
O3	0.34432 (18)	1.1322 (4)	0.43176 (16)	0.0417 (6)	
N1	0.25815 (18)	0.9487 (3)	0.12794 (16)	0.0218 (5)	
C1	0.0437 (2)	0.9680 (4)	0.1309 (2)	0.0201 (5)	
C2	0.1346 (2)	0.9582 (4)	0.06419 (19)	0.0195 (5)	
C3	0.0870 (2)	0.9607 (4)	−0.06108 (19)	0.0211 (5)	
H3	0.1438	0.9574	−0.1029	0.025*	
C4	0.3199 (2)	0.9490 (4)	0.2607 (2)	0.0251 (5)	
H4	0.2809	0.8534	0.2977	0.030*	
C5	0.4542 (2)	0.8929 (4)	0.2793 (2)	0.0297 (6)	
H5A	0.5139	0.9458	0.3559	0.036*	
H5B	0.4635	0.7552	0.2815	0.036*	
C6	0.4758 (2)	0.9750 (5)	0.1691 (2)	0.0291 (6)	
H6A	0.5411	0.9044	0.1508	0.035*	
H6B	0.5013	1.1078	0.1830	0.035*	
C7	0.3489 (2)	0.9556 (4)	0.0660 (2)	0.0236 (5)	
H7A	0.3324	1.0638	0.0101	0.028*	
H7B	0.3454	0.8397	0.0190	0.028*	
C8	0.3099 (2)	1.1413 (4)	0.3104 (2)	0.0261 (6)	
C9	0.3358 (4)	1.3085 (7)	0.4894 (3)	0.0656 (12)	
H9A	0.3767	1.4069	0.4595	0.098*	
H9B	0.3772	1.2966	0.5778	0.098*	
H9C	0.2476	1.3409	0.4698	0.098*	

### Atomic displacement parameters (Å^2^)

**Table:**

	*U*^11^	*U*^22^	*U*^33^	*U*^12^	*U*^13^	*U*^23^
O1	0.0265 (8)	0.0356 (11)	0.0146 (7)	−0.0001 (8)	0.0063 (6)	0.0006 (8)
O2	0.0369 (10)	0.0308 (11)	0.0359 (10)	−0.0014 (9)	0.0174 (8)	−0.0048 (9)
O3	0.0396 (11)	0.0594 (14)	0.0204 (9)	0.0108 (11)	0.0040 (8)	−0.0119 (10)
N1	0.0203 (9)	0.0265 (11)	0.0177 (9)	0.0016 (9)	0.0057 (7)	−0.0024 (9)
C1	0.0233 (10)	0.0196 (11)	0.0180 (9)	0.0033 (10)	0.0082 (8)	0.0030 (10)
C2	0.0225 (10)	0.0185 (11)	0.0174 (10)	0.0002 (11)	0.0071 (8)	−0.0006 (10)
C3	0.0232 (10)	0.0234 (11)	0.0186 (10)	−0.0013 (10)	0.0097 (8)	−0.0033 (10)
C4	0.0224 (11)	0.0328 (14)	0.0175 (10)	0.0019 (12)	0.0041 (8)	−0.0005 (11)
C5	0.0265 (12)	0.0330 (14)	0.0256 (12)	0.0064 (11)	0.0046 (9)	−0.0003 (11)
C6	0.0219 (11)	0.0326 (14)	0.0309 (12)	−0.0004 (11)	0.0072 (9)	−0.0011 (12)
C7	0.0219 (11)	0.0255 (12)	0.0247 (10)	0.0023 (11)	0.0100 (8)	−0.0003 (11)
C8	0.0175 (11)	0.0380 (15)	0.0215 (11)	0.0021 (11)	0.0055 (8)	−0.0045 (11)
C9	0.064 (2)	0.088 (3)	0.0364 (16)	0.017 (2)	0.0078 (15)	−0.034 (2)

### Geometric parameters (Å, °)

**Table:**

O1—C1	1.238 (3)	C4—C5	1.530 (3)
O2—C8	1.203 (4)	C4—H4	1.0000
O3—C8	1.341 (3)	C5—C6	1.525 (4)
O3—C9	1.453 (5)	C5—H5A	0.9900
N1—C2	1.345 (3)	C5—H5B	0.9900
N1—C4	1.466 (3)	C6—C7	1.528 (3)
N1—C7	1.473 (3)	C6—H6A	0.9900
C1—C3^i^	1.426 (3)	C6—H6B	0.9900
C1—C2	1.518 (3)	C7—H7A	0.9900
C2—C3	1.377 (3)	C7—H7B	0.9900
C3—C1^i^	1.426 (3)	C9—H9A	0.9800
C3—H3	0.9500	C9—H9B	0.9800
C4—C8	1.516 (4)	C9—H9C	0.9800
			
C8—O3—C9	114.4 (3)	C4—C5—H5B	110.9
C2—N1—C4	126.92 (18)	H5A—C5—H5B	109.0
C2—N1—C7	120.87 (18)	C5—C6—C7	103.91 (19)
C4—N1—C7	111.96 (18)	C5—C6—H6A	111.0
O1—C1—C3^i^	121.5 (2)	C7—C6—H6A	111.0
O1—C1—C2	120.2 (2)	C5—C6—H6B	111.0
C3^i^—C1—C2	118.3 (2)	C7—C6—H6B	111.0
N1—C2—C3	121.90 (19)	H6A—C6—H6B	109.0
N1—C2—C1	119.72 (19)	N1—C7—C6	104.46 (19)
C3—C2—C1	118.4 (2)	N1—C7—H7A	110.9
C2—C3—C1^i^	123.07 (19)	C6—C7—H7A	110.9
C2—C3—H3	118.5	N1—C7—H7B	110.9
C1^i^—C3—H3	118.5	C6—C7—H7B	110.9
N1—C4—C8	109.8 (2)	H7A—C7—H7B	108.9
N1—C4—C5	102.97 (18)	O2—C8—O3	124.5 (3)
C8—C4—C5	113.3 (2)	O2—C8—C4	126.0 (2)
N1—C4—H4	110.2	O3—C8—C4	109.5 (2)
C8—C4—H4	110.2	O3—C9—H9A	109.5
C5—C4—H4	110.2	O3—C9—H9B	109.5
C6—C5—C4	104.1 (2)	H9A—C9—H9B	109.5
C6—C5—H5A	110.9	O3—C9—H9C	109.5
C4—C5—H5A	110.9	H9A—C9—H9C	109.5
C6—C5—H5B	110.9	H9B—C9—H9C	109.5
			
C4—N1—C2—C3	178.7 (3)	C7—N1—C4—C5	−17.7 (3)
C7—N1—C2—C3	4.9 (4)	N1—C4—C5—C6	32.4 (3)
C4—N1—C2—C1	−0.9 (4)	C8—C4—C5—C6	−86.2 (2)
C7—N1—C2—C1	−174.7 (2)	C4—C5—C6—C7	−35.3 (3)
O1—C1—C2—N1	4.5 (4)	C2—N1—C7—C6	170.6 (2)
C3^i^—C1—C2—N1	−174.4 (2)	C4—N1—C7—C6	−4.1 (3)
O1—C1—C2—C3	−175.1 (2)	C5—C6—C7—N1	24.3 (3)
C3^i^—C1—C2—C3	6.1 (3)	C9—O3—C8—O2	2.6 (4)
N1—C2—C3—C1^i^	179.1 (3)	C9—O3—C8—C4	−178.7 (2)
C1—C2—C3—C1^i^	−1.4 (3)	N1—C4—C8—O2	−13.2 (3)
C2—N1—C4—C8	−71.0 (3)	C5—C4—C8—O2	101.3 (3)
C7—N1—C4—C8	103.3 (2)	N1—C4—C8—O3	168.10 (19)
C2—N1—C4—C5	168.0 (2)	C5—C4—C8—O3	−77.4 (2)

Symmetry codes: (i) −*x*, *y*, −*z*.

### Hydrogen-bond geometry (Å, °)

**Table:**

*D*—H···*A*	*D*—H	H···*A*	*D*···*A*	*D*—H···*A*
C3—H3···O2^ii^	0.95	2.56	3.400 (3)	147
C5—H5b···O1^iii^	0.99	2.54	3.407 (3)	146
C9—H9b···O1^iv^	0.98	2.38	3.186 (4)	139

Symmetry codes: (ii) −*x*+1/2, *y*−1/2, −*z*; (iii) *x*+1/2, *y*−1/2, *z*; (iv) −*x*+1/2, *y*+1/2, −*z*+1.
